# Quantitative insight into the combinatorial interactions within MYB-BHLH-WD complex in *Arabidopsis thaliana*

**DOI:** 10.17912/micropub.biology.000293

**Published:** 2020-08-19

**Authors:** Mikiya Umeyama, Kengo Morohashi

**Affiliations:** 1 Faculty of Science and Technology, Department of Applied Biological Science, Tokyo University of Science, 2641 Yamazaki, Noda, Chiba 278-8510, Japan

**Figure 1.**
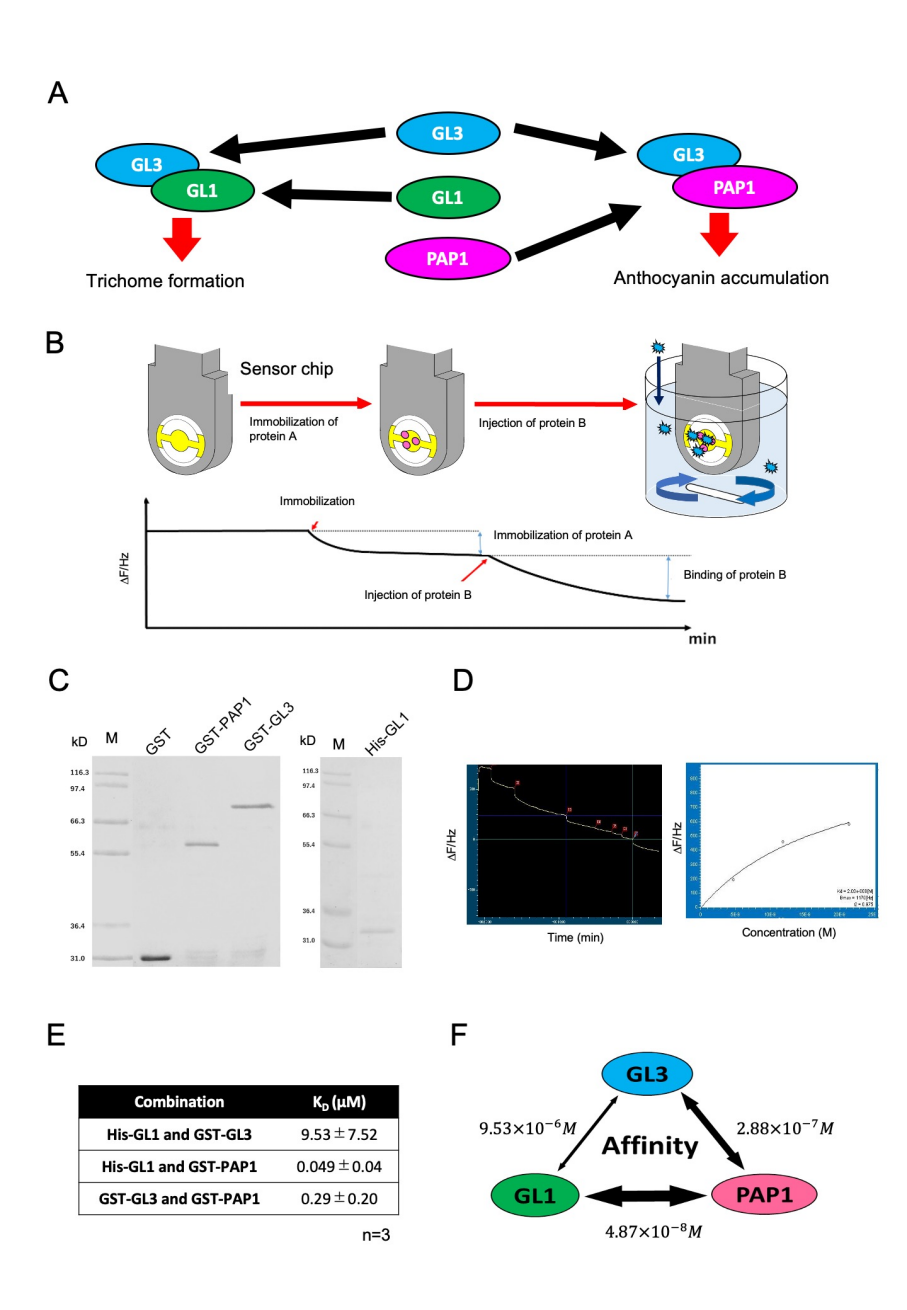
Affinity measurement of GL3, GL1 and PAP1 proteins. (A) Schematic representation of MBW complex that consists of GL3, GL1 and PAP1. GL3-GL1 and GL3-PAP1 complex regulate downstream genes involved in trichome formation and anthocyanin accumulation, respectively. WD-repeat protein is not shown in this figure. (B) Schematic representation of QCM. (C) Coomassie brilliant blue staining gels. GST, GST-PAP1, GST-GL3 and His-GL1 were loaded. (D) Representative result of output of QCM analysis. Left panel shows a result of real-time monitoring of frequency of a quartz crystal. Injection points of proteins samples are indicated as red boxes. Right panel shows a non-linear fitting plot on the difference of frequency of a quartz crystal. (E) A table of *K_D_* values. *K_D_* values are shown as averages from three independent quantification. (F) Summary of affinities between GL3, GL1 and PAP1.

## Description

Plants protect themselves from different environmental stresses, and the responses are governed by multiple transcription factors (TFs) via complex forms. Plants evolutionally conserve a heteromeric complex, comprised of MYB, basic helix-loop-helix (bHLH), and WD-repeat proteins (MBW) (Albert *et al.* 2014; Xu *et al.* 2015; Lloyd *et al.* 2017). The combination of MYB and bHLH in MBW is responsible for a wide range of biological events, while the variation of WD-repeat protein is low. In *Arabidopsis thaliana*, GL3, bHLH, forms a complex with GL1, MYB, and the GL3-GL1 complex generates a trichome in epidermal cells, which is believed to protect the plant body from insects. GL3 also forms a complex with PAP1, another MYB, and the GL3-PAP1 complex regulates the synthesis of anthocyanin which protects against UV and strong light (Fig 1A). Because MBW consists of various MYBs, a competition between MYB to bHLH impacts the activity and the regulation of gene expression (Zhang and Hülskamp, 2019). Thus, the affinity between MYB and bHLH proteins are essential for understanding the mechanism underlying the combinatorial regulation of MBW complex (Lloyd *et al.* 2017). However, little is known about the quantitative evaluation of affinities between MYBs and bHLH. To acquire affinity information of MBW in a quantitative manner, we focused on GL3, GL1, and PAP1. Because these proteins are expressed in an epidermal cell at early leaf development, GL3, GL1, and PAP1 would integrally impact the trichome formation and anthocyanin biosynthesis via competitive interactions. We quantified the dissociation constants of these three proteins using a quartz crystal microbalance (QCM). QCM is a biosensor to monitor in real time an interaction that occurred on a sensor chip by a frequency of a quartz crystal resonator (Fig 1B) (Fee, 2013; Johannsmann, 2015). We purified the recombinant proteins of GST-tagged GL3, His-tagged GL1, and GST-tagged PAP1, which were expressed in bacteria and confirmed using SDS electrophoresis (Fig 1C). Purified GL1 and GL3 were separately immobilized on the surface of two sensor chips. The immobilization was observed as a difference of frequency. Following the confirmation of stable immobilization, we proportionally added the other proteins, GL3 or PAP1, in a cuvette, because the difference of frequency represents the amount of correlated proteins. Dissociation constants were measured by applying nonlinear fit on Langmuir formula (Fig 1D), resulting in the dissociation constants (*K_D_*) of GL3 and GL1, GL3 and PAP1, and PAP1 and GL1, which were 9.53 ± 7.52 µM, 0.29 ± 0.20 µM, and 0.049 ± 0.04 µM, respectively (average of three independent experiments) (Fig 1E). Previously, the *K_D_* values between EGL3, GL3, and PAP2 were described using a different system from ours, wherein the range of *K_D_* between bHLH and MYBs was 3.5–7.5 µM (Nemie-Feyissa *et al.* 2015). Since we can not rule out that the GL3-PAP1 interaction is due to GST dimerization, our conclusion should be considered as preliminary. Nevertheless, these *K_D_* values are in a similar range with the GL3-GL1 interaction in our study. It is notable that our result shows that the affinity between GL1 and PAP1 was 100-fold higher than that between GL3 and GL1 (Fig. 1F). These results propose that the competitive regulation in the MBW complex should be considered not only between bHLH and MYB but also between MYBs.

## Methods

Construction of expression vectors

CDS regions of GL1 (At3g27920), GL3 (At5g41315), and PAP1 (At1g56650) were amplified by PCR with Prime STAR (TAKARA Bio, Shiga, Japan) from the pDONAR vectors containing the GL1, GL3, and PAP1 CDS provided by Nobutaka Mitsuda (National Institute of Advanced Industrial Science and Technology) with following oligo DNA primers, respectively. MU_GL1_F, 5’-AATGGGTCGCGGATCCGAAATGAGAATAAGGAGAAGAGATG-3’; MU_GL1_R, 5’-TGTCGACGGAGCTCGAATTAAGGCAGTACTCAACATCACC-3’; MU_GL3_F, 5’-GCCCCTGGGATCCCCGGAAATGGCTACCGGACAAAACAGAA-3’; MU_GL3_R, 5’-TCGAGTCGACCCGGGAATTTCCTGATGATGATGACGATGA-3’; MU_PAP1_F, 5’-GCCCCTGGGATCCCCGGAAATGGAGGGTTCGTCCAAAGGG-3’; MUPAP1_R, 5’-TCGAGTCGACCCGGGAATTATCAAATTTCACAGTCTCTCC-3’. The DNA fragment was cleaved by EcoR1 and was cloned into the Shrimp Alkaline phosphatase treated pGEX6P-1 vector using HiFi DNA Assembly Master Mix (NEB, Ipswich, Massachusetts). For GL1, construction was also created in the pET21a-d vector; these constructs were introduced into Rozetta 2 (DE3) by the heat shock transformation

Purification of recombinant proteins

Single colonies of Rozetta 2 (DE3) transformed with GL1-pGEX6P-1, GL3-pGEX6P-1, PAP1-pGEX6P-1, and GL1-pET21a-d were grown in 50 mg/L carbenicillin containing LB liquid medium at 37˚C. 3 ml of pre-cultured cells was added to 300 ml of LB liquid medium containing 50 mg/L carbenicillin and cultured at 37°C until the OD600 reached 0.6. IPTG (isopropyl b-D-1-thiogalactopyranoside) was added at a final concentration of 1 mM, and induction was carried out at 20°C for 20 hours. After induction, the cells were collected for 15 minutes at 3500 rpm and washed once with 50 ml of PBS. In the case of GST and GST-fused proteins, cells were suspended in 30 ml of ice-cold GST buffer ((20 mM Tris-HCl (pH 8.0), 1 mM EDTA, 1% TritonX, 1 mM PMSF, 5 mM DTT, 1 mM PMSF, 1 mM protease inhibitor cocktail), 3 ml of 10 mg/ml lysozyme was added and incubated for 30 minutes at 37° C. Sonication was performed for 30 seconds for four times on ice. 300 μl of Triton X-100 was added, and the mixture was stirred at 4° C for 15 minutes by a rotator, followed by centrifugation at 9,000 rpm for 15 min at 4°C. 1 mL of Glutathione-Sepharose 4B(GE), which washed twice with ice-cold GST buffer, was added to the supernatant, and the mixture was stirred at 4°C overnight by a rotator. After centrifugation at 5,000 rpm for 5 min at 4°C, the supernatant was removed, and the beads were washed three times with 10 ml of ice-cold GST buffer (1 mM DTT), and proteins were eluted by 2.0 ml of High Salt Elution Buffer (20 mM Reduced Glutathione, 100 mM Tris-HCl (pH 8.0), 120 mM NaCl). In the case of His-GL1, precipitated induced cells were resuspended to 30 ml of ice-cold TKET buffer (10 mM Tris-HCl pH 7.5, 100 mM KCl, 0.1 mM EDTA, 100 mM NaCl, 0.05% Triton X100, 1 mM PMSF, 1 mM protease inhibitor cocktail) containing a final concentration of 10 mM Imidazole. 3 ml of 10 mg/ml lysozyme was added, and the mixture was incubated at 37°C for 30 minutes. The mixture was sonicated for 30 seconds for four times on ice. 300 ul of Triton X-100 was added, and the mixture was stirred at 4° C for 15 minutes by a rotator. After centrifugation at 9,000 rpm for 15 min at 4°C, 1 ml of Ni-sepharose 6FF(GE), which was washed twice with ice-cold TKET buffer (10 mM Imidazole), was added to the supernatant, and the mixture was stirred at 4°C overnight by a rotator. After centrifugation at 5,000 rpm for 5 min at 4°C, the supernatant was removed, and the beads were washed three times with 10 ml of ice-cold TKET buffer (10 mM Imidazole). Proteins were eluted with ice-cold TKET buffer (300 mM Imidazole).

Protein sample preparation

10 ul SDS sample buffer (125 mM 0.5 M Tris-HCl buffer (pH 6.8), 4% SDS, 20% glycerol, 0.01% BPB, 10% 2-mercaptoethanol) was added to 10 ul of purified protein. Samples were boiled for 5 minutes at 90°C. Then, SDS-PAGE was performed by a 10% acrylamide gel, followed by CBB staining. After confirmation of a single band, the buffer of purified proteins was exchanged with PBS (1,370 mM NaCl, 27 mM KCl, 81 mM Na2HPO4·12H2O, 14.7 mM KH2PO4) using Amicon Ultra (Merck, Kenilworth, New Jersey). The protein concentration was measured with Pierce BCA Protein Assay Kit (Thermo Fisher Scientific, Waltham, Massachusetts), and ultrafiltration was performed until the concentration reached 750 ug/ml or more.

Dissociation constant measurement

Immobilization was performed according to the manufactural protocol of the immobilization Affinix kit (Initium Inc. Tokyo, Japan). Shortly, the sensor chip was initialized. The electrode was carefully rubbed with a cotton swab soaked in 1% SDS, washed with running DW water, and dried by blowing air. Next, 2 μl of piranha solution (concentrated sulfuric acid: 30% H2O2=3:1) prepared in a glass container was added only on the gold film of the sensor chip, and the mixture was allowed to stand for 5 minutes. It was rewashed with running water of DW and dried by blowing air. This step was performed twice in total. An appropriate attached buffer was selected and adjusted to 100 ul (Buffer 5 μl, 200 ug/ml protein solution 50 ul, DW 45 ul). After stuffing the edges of the plastic petri dish with moistened paper and arranging the ceramics sensors, 50 μl of the SAM solution was mounted on the sensor gold film, shielded with aluminum foil, and allowed to stand at room temperature for 1 hour. After drying by blowing air, 50 μl of a solution obtained by mixing equal ratio of NHS solution and EDC solution was mounted on the gold film, shielded from light, and allowed to stand at room temperature for 15 minutes. After washing with DW running water and air, the frequency of quartz was measured. 100 µl of the prepared protein solution was mounted on the gold film. After shielding from light and standing at room temperature for 1 hour, the air was blown to dry it, and then the frequency of quartz was measured again. Washing and air were repeated until the frequency of quartz was lower than the measured value by 1000 Hz. 100 μl of ethanolamine was mounted, and protected from light, and allowed to stand at room temperature for 10 minutes. A frequency was measured again and used as the final fixed amount.

The protein-immobilized sensor was attached to Affinix Q (Initium Inc. Tokyo, Japan) and placed in a glass cell containing 8 ml of PBS to stabilize it. After the output of the frequency of quartz become stable, 1 to 32 ul of the protein solution was injected. Protein solutions with different volumes were injected multiple times. Then, the dissociation constant was calculated by AQUA (Initium Inc. Tokyo, Japan). The measurement conditions were set at a set temperature of 25°C, a stirrer rotation speed of 1,000 rpm, and a data acquisition speed was 1 second. The experiments were repeated at least three times by using different sensor chips in each measurement of interaction.
